# Comparison between transpancreatic sphincterotomy and needle-knife fistulotomy in difficulty biliary access, a retrospective study in Taiwan

**DOI:** 10.1186/s12876-020-01323-x

**Published:** 2020-06-19

**Authors:** Kai-Shun Liang, Chieh-Chang Chen, Wei-Chih Liao, Yu-Ting Kuo, Liang-Wei Tseng, Wen-Tsung He, Hsiu-Po Wang

**Affiliations:** 1 Department of Internal Medicine, Fu Jen Catholic University Hospital, New Taipei City, Taiwan; 2 Division of Gastroenterology and Hepatology, Department of Internal Medicine, National Taiwan University Hospital, Taipei, Taiwan; 3 Department of Internal Medicine, College of Medicine, National Taiwan University, Taipei, Taiwan; 4 Integrated Diagnostics and Therapeutics, National Taiwan University Hospital, National Taiwan University, Taipei, Taiwan

**Keywords:** Transpancreatic sphincterotomy, Needle knife fistulotomy, Endoscopic retrograde cholangiopancreaticography, Complication, Success rate

## Abstract

**Background:**

Selective deep biliary cannulation is the first and the most important step before further biliary therapy. Transpancreatic sphincterotomy (TPS), and needle knife fistulotomy (NKF) were commonly used in patients with difficult cannulation, but few studies compare the outcome between TPS and NKF.

**Methods:**

A total of 78 patients who met the criteria of difficult cannulation in the National Taiwan University hospital from October 2015 to October 2017 were retrospectively reviewed. Their baseline demographics, success rate of biliary cannulation, and the rate of adverse events were assessed.

**Results:**

31 patients and 47 patients underwent TPS and NKF for difficult biliary access, respectively. The characteristics of the 2 groups were similar, but patients in TPS group had more frequent pancreatic duct cannulation. Bile duct cannulation was successful in 23 patients (74.2%) in the TPS group and 39 (83.0%) in the NKF group (*P* = 0.34). There was no difference between the TPS and NKF in the rate of adverse events, including post-ERCP pancreatitis (PEP) (16.1% vs. 6.4%, *p* = 0.17), and hemorrhage (3.2% vs. 8.5%, *p* = 0.35). No perforation occurred.

**Conclusions:**

Both TPS and NKF have good biliary access rate in patient with difficult cannulation. TPS has acceptable successful rate and similar complication rate, compared with NKF.

## Background

Endoscopic retrograde cholangiopancreaticography (ERCP) is nowadays a widely-used technique for managing pancreatobiliay diseases. Selective deep biliary cannulation is the first and the most important step for further therapeutic biliary interventions. However, deep biliary cannulation is not achieved by initial attempts in 10–20% of patients with a native major papilla [1]. Difficult cannulation increases the risk of post-ERCP adverse events, particularly post-ERCP pancreatitis (PEP), post-ERCP cholangitis and perforation. Various techniques has developed to overcome difficult cannulation, including transpancreatic sphincterotomy (TPS), double guidewire technique, needle knife papillotomy (NKP), and needle knife fistulotomy (NKF).

The needle-knife technique was first described in the early 1980s and has been widely performed nowadays [2]. The needle-knife technique includes a precut papillotomy which the incision starts from the papillary orifice and a precut fistulotomy, which on the other hand, the incision starts above the papillary orifice. Mavrogiannis et al. [3] found a significantly lower PEP rate for precut fistulotomy but a similar success rate at the initial bile duct cannulation between needle-knife fistulotomy and needle-knife precut papillotomy. The TPS technique was first described in 1995. In TPS, after superficial or deep cannulation of the pancreatic duct is achieved, a sphincterotome is used to cut the septum between bile and pancreatic ducts along the direction of 11 o’clock to 12 o’clock [4]. TPS is less technically demanding and easier to control the depth of cutting [5]. When the pancreatic duct is repeatedly cannulated in patients with difficult biliary access, TPS may be a simple way to find the way to bile duct. Two randomized control trials (RCTs) showed that TPS had higher primary success rates than NKP [5, 6]. A RCT demonstrated that NKF had a lower risk of PEP than NKP [3], However, there were few studies comparing the efficacy and adverse event rate between TPS and NKF. The present study aims to compare the rates of successful cannulation and adverse events between TPS and NKF in patients with difficult biliary access..

## Methods

### Study design

We searched our prospectively maintained ERCP database for patients who underwent transpancreatic sphincterotomy (TPS) or needle knife fistulotomy (NKF) for difficult biliary access in National Taiwan University Hospital from October 2015 to October 2017. Those who failed the first attempt of cannulation for more then 10 min and then received TPS or NKF were enrolled in. We retrieved patient characteristics including gender, age, and indication of ERCP. Procedural information was also collected, such as endoscopic findings, total number of pancreatic duct cannulations, post-cannulation procedures and measures for PEP prophylaxis. Finally, we recorded the success rate as the primary outcome and adverse events of the patients as the secondary outcome by following up the patients’ clinical condition and blood tests such as levels of hemoglobin, serum total bilirubin and amylase/lipase.

### Procedures

All the procedure during the study period were performed by five experienced endoscopists, who performed more than 100 therapeutic ERCP per year. Endoscopic retrograde cholangiopancreaticography and further intervention were performed with a standard side-view duodenoscope (TJF260, Olympus, Tokyo, Japan). The bile duct cannulation was attempted firstly with catheter with a inserted guidewire. When encountering failure of first attempt for biliary cannulation, endoscopists may choose TPS, NKF or double guidewire as salvage method for achieving successful biliary access by their clinical judgement, and there was no definite or consensual strategies for difficult biliary cannulation among endoscopists in our hospital. In patient undergoing TPS, TPS was performed as Goff reported [7]; in short, after cannulation of the pancreatic duct was achieved, a triple-lumen sphincterotome (V KD-V411M-0730, Olympus, Tokyo, Japan or TRUEtome cut wire 4.4F × 30 mm (Boston Scientific Taiwan, Taipei, Taiwan) on a guidewire was used to cut the septum between bile and pancreatic ducts along the direction of 11 o’clock to 12 o’clock. After this, the sphincterotomy was extended to expose the biliary lumen and the biliary duct could be cannulated [8]. In patient with NKF, a needle-knife with MicroKnife XL 5.5F (Boston Scientific Taiwan, Taipei, Taiwan) and an ERBE electrosurgical generator were used to perform a stepwise incision of the mucosa above the papillary orifice followed by downward cut until the underlying biliary sphincter was visualized [8].

### Definitions of complications

We followed the definition of post-ERCP pancreatitis (PEP) according to a consensus from Cotton et all [9]., which was originally defined as “clinical pancreatitis with amylase at least three times normal at more than 24 hours after the procedure, requiring hospital admission or a prolongation of planned admission”. The definition of significant post-ERCP hemorrhage was defined as clinical (not just endoscopic) evidence of bleeding such as melena or hematemesis according to the same consensus [9]. We also record post-ERCP hemorrhage judged by endoscopy as “endoscopically bleeding”. Perforation was referred to as document by any radiographic studies. Cholangitis was defined as fever with temperature more than 38 °C because of biliary source without evidence of other concomitant infections [10].

### Statistical analysis

Statistical analyses were performed using Stata 13.0 software (Stata Corp LP, College Station, TX, United States). Statistical analysis was performed using chi-squared tests for categorical data and the Student’s *t* test for continuous data. Mann-Whitney U test was used for post- and pre-ERCP amylase/lipase levels and number of pancreatic duct cannulation. *P* value of < 0.05 was regarded as statistically significant. Univariable analyses were performed to assess the outcomes and adverse events of ERCP in patients who underwent TPS or NKF. We also used multivariable logistic regression to assess the association between PEP and TPS or NKF while adjusting for age, gender, number of pancreatic duct cannulation, endoscopic papillary balloon dilatation (EPBD) and PEP prophylaxis.

## Results

**Fig. 1:**
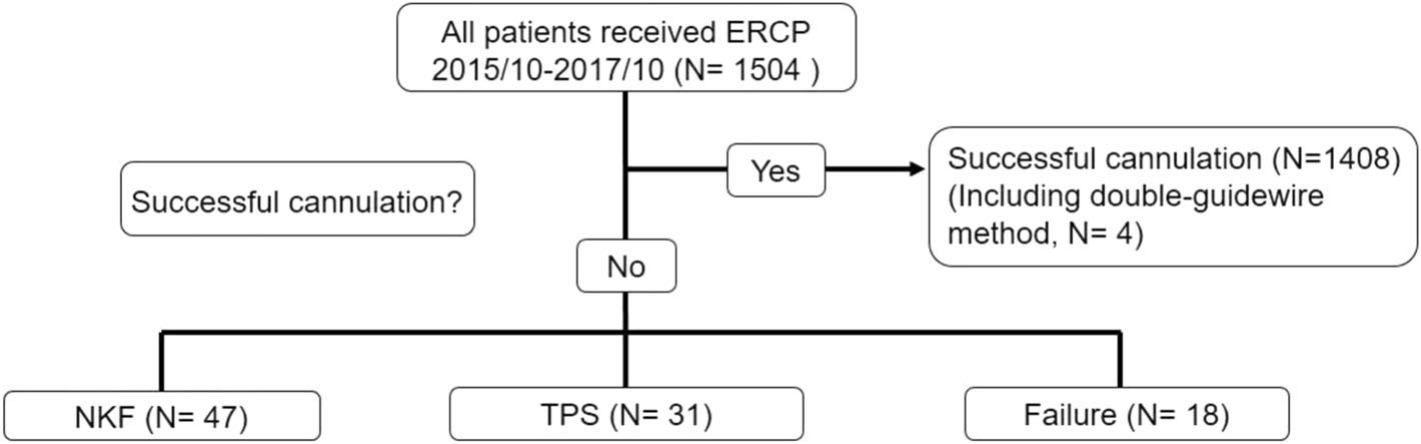
Flow chart of the study design. ERCP: Endoscopic retrograde cholangiopancreaticography, TPS: Transpancreatic sphincterotomy, NKF: needle knife fistulotomy

From October 2015 to October 2017, 1504 patients underwent ERCP in National Taiwan University Hospital, and successful cannulation was achieved in 1408 patients. Among them, two patients underwent double-guidewire method. Eighteen patients with deep CBD cannulation failure (Fig. 1). Among 78 patients included for analyses, 31 patients received TPS and 47 ones received NKF (Table 1). All cases of subject were with naive papilla. The mean age was 69.6 years old. The major indications of ERCPs included 38 patients (48.7%) of bile duct stone, and 27 patients (34.7%) of malignant bile duct obstruction. Overall success rate of deep cannulation was 79.5% (62 patients). 5 (6.4%) patients complicated with bleeding, 8 (10.3%) patient had post-ERCP pancreatitis and 3 (2.6%) patients had post-ERCP cholangitis. None of them had perforation.

**Table 1:** Characteristics of cases with difficult cannulation

Item, n(%)	No/Percentage
Gender (M: F)	41: 37
Age, mean (SD)	69.6 (13.5)
ERCP indication	
CBD stone	38 (48.7%)
Pancreatic tumor/cancer	23 (29.5%)
Cholangiocarcinoma	4 (5.2%)
Others	13 (16.6%)
Procedures	
NKF	47 (60.3%)
TPS	31 (39.7%)
No. of deep cannulation achieved	62 (79.5%)
Complications	
Bleeding	5 (6.4%)
PEP	8 (10.3%)
Acute cholangitis	2 (2.6%)
Perforation	0 (0%)

*TPS* Transpancreatic sphincterotomy, *NKF* needle knife fistulotomy, *ERCP* Endoscopic retrograde cholangiopancreaticography, *PEP* post-ERCP pancreatitis, *CBD* common bile duct

Table 2 presented the characteristics of the TPS and NKF group. Patients in TPS group had more pancreatic duct cannulation, compared with patients in NKF group. More than half of patients (16/31) received three to six times of pancreatic duct cannulation during ERCP. 23 (74.2%) patients in TPS group had successful bile duct cannulation, while 39 (83.0%) patients in NKF group access bile duct successfully. There was no significant difference regarding the bile duct cannulation rate between two groups (*p* = 0.34). Moreover, three patients achieved successful bile duct cannulation after NKF, though encountering failure during TPS in the beginning. These three patients were not counted as successful bile duct cannulation.

**Table 2:** Comparison of NKF and TPS groups

	TPS (***n*** = 31)	NKF (***n*** = 47)	p
Male, n (%)	14 (45.2)	27 (57.5)	0.28
Age, mean (SD)	71.2 (14.5)	68.5 (12.9)	0.39
ERCP indication			
CBD stone, n (%)	19 (61.3)	20 (42.6)	0.11
Pancreatic neoplasm, n (%)	8 (25.8)	15 (31.9)	0.56
Cholangiocarcinoma, n (%)	2 (6.5)	2 (4.3)	0.67
Others (CP, other malignancy), n (%)	2 (6.5)	10 (21.3)	0.08
Pancreatic duct – cannulation			
P – cannulation < 3, n (%)	6 (19.4)	43 (91.5)	< 0.001
P – cannulation ≥3, n (%)	16 (51.6)	4 (8.5)	< 0.001
P – cannulation > 5, n (%)	6 (19.4)	0 (0)	0.002
P – cannulation > 8, n (%)	2 (9.7)	0 (0)	0.03
P – cannulation, median (IQR)	4.5 (3)	0 (0)	0.002
Endoscopic finding			
Diverticulum, n (%)	6 (23.1)	4 (8.5)	0.03
Deep cannulation, n (%)			
Success	23 (74.2)	39 (83.0)	0.34
TPS + NKF	3 (9.7)		
Failure	4 (12.9)	8 (17)	
Post – cannulation procedure, n (%)			
EPLBD	3 (9.7)	5 (10.6)	0.891
EPBD	7 (22.6)	5 (10.6)	0.15
ERBD (plastic stent)	8 (25.8)	18 (38.3)	0.25
metallic stent	0 (0)	6 (12.7)	0.04
Lithotripsy	14 (45.2)	15 (31.9)	0.236
PEP prophylaxis, n (%)			
Nil	13 (41.9)	34 (72.3)	0.007
P - stenting	4 (12.9)	6 (12.8)	0.99
Gabexate Mesilae	12 (38.7)	5 (10.6)	0.003
NSAIDs	0	2 (4.26)	0.24
Gabexate Mesilae + NSAIDs	5 (16.1)	3 (6.4)	0.17

*TPS* Transpancreatic sphincterotomy, *NKF* needle knife fistulotomy, *ERCP* Endoscopic retrograde cholangiopancreaticography, *PEP* post-ERCP pancreatitis, *NKP* needle knife papillotomy, *CBD* common bile duct, *EPBD* endoscopic papillary balloon dilatation (balloon diameter less than 12 mm), *EPLBD* endoscopic papillary large balloon dilatation (large-diameter balloons (12–20 mm)), *ERBD* endoscopic retrograde biliary drainage, *NSAID* Non-Steroidal Anti-Inflammatory Drug, *CP* chronic pancreatitis

For the prophylaxis of PEP, four (12.9%) patients in TPS group and six (12.8%) patients in NKF group received prophylactic pancreatic stenting. Significantly more patients (twelve, 38.7%) in TPS group received continuous gabexate mesilate infusion after ERCP, comparing with five (10.6%) patients in NKF group. Five (16.1%) patients in TPS group and three (6.4%) patients in NKF group received combination of gabexate mesilate infusion and diclofenac rectal suppository. Two (4.26%) patients in NKF group received diclofenac sodium rectally for PEP prophylatic treatment. More patients in NKF group received no PEP prophylaxis than in TPS group (72.3% vs. 41.9%, respectively, *p* = 0.007).

Table 3. summarized the adverse events after ERCP. The overall adverse events after ERCP were similar in both groups (TPS vs. NKF, 19.3% vs. 19.1%, respectively, *p* = 0.99). There were no significant difference between TPS and NKF group in post-ERCP bleeding, acute cholangitis and perforation (19.3% vs. 12.8, 0% vs. 4.3, 0% vs 0%, respectively). Patients in TPS group had higher Post-ERCP amylase level than patients in NKF group (median level: 155 U/L vs. 62 U/L, respectively, *p* = 0.01). Five (16.1%) patients in TPS group developed PEP, while three (6.4%) patients in NKF group had PEP (*p* = 0.17). There were six patients (three in TPS group and three in NKS group) of PEP in successful cannulated patients. The total incidence of PEP in successful cannulated patients was 9.7%(6/62), with 13.0% in TPS group (3/23) and 7.7% in NKF group (3/39) respectively. All patients had PEP were mild in severity, and the details of the PEP patients was summarized in Table 4.

**Table 3:** Outcomes of NKF and TPS groups

	TPS(n = 31)	NKF(n = 47)	p
Post-ERCP amylase level > 3 ULN, n(%)	9 (29.0)	5 (11.9)	0.07
Post-ERCP lipase level > 3 ULN, n(%)	13 (41.9)	11 (25.0)	0.14
Post-ERCP amylase level, median (IQR)	155 (260)	62 (103)	0.01
Post-ERCP lipase level, median (IQR)	148 (590)	97 (209.5)	0.21
PEP, n (%)	5 (16.1)	3 (6.4)	0.17
Mild	5 (16.1)	3(6.4)	
Moderate	0 (0)	0 (0)	
Severe	0 (0)	0 (0)	
Bleeding, n (%)			
All bleeding	1 (3.2)	4 (8.5)	0.35
Significant bleeding	1 (3.2)	1 (2.1)	0.76
Acute cholangitis, n (%)	0 (0)	2 (4.3)	0.25
Perforation, n (%)	0 (0)	0 (0)	
Total complication events (endoscopically bleeding + perforation + PEP + cholangitis), n (%)	6 (19.3)	9 (19.1)	0.99
Total complication events (significant bleeding + perforation + PEP + cholangitis), n (%)	6 (19.3)	6 (12.8)	0.51

*TPS* Transpancreatic sphincterotomy, *NKF* needle knife fistulotomy, *ERCP* Endoscopic retrograde cholangiopancreaticography, *PEP* post-ERCP pancreatitis,

**Table 4:** Details of PEP patients

No	Gender^a^	Age	Indication	Cannulation methods	Pancreatic cannulation	Biliary access	EPBD	Prophylaxis	Severity of PEP
1	2	50- 59	CBD stone	NKF	0	Yes	Y	none	Mild
2	2	60- 69	Pancreatic cancer	NKF	0	Yes	Y	none	Mild
3	2	80- 89	Pancreas head tumor	NKF	0	Yes	N	none	Mild
4	1	60- 69	CBD stone	TPS	4	Yes	Y	NSAID + gabexate	Mild
5	2	60- 69	Cholangiocarcinoma	TPS	12	No	N	none	Mild
6	2	30- 39	CBD stone	TPS	5	Yes	N	NSAID + gabexate	Mild
7	1	40- 49	Pancreatic cancer	TPS	12	No	N	P stent + NSAID + gabexate	Mild
8	1	60- 69	CBD stone	TPS	No	Yes	Y	NSAID + gabexate	Mild

^a^*EPBD* endoscopic papillary balloon dilatation, *TPS* Transpancreatic sphincterotomy, *NKF* needle knife fistulotomy, *NSAID* Non-Steroidal Anti-Inflammatory Drug

Since both groups had similar adverse events after ERCP, we tried to investigate the factors associated with PEP. Univariate analysis (Table 5.) showed that younger than 65-year-old diclofenac sodium +/− gabexate mesilate treatment after ERCP are statistically significant patient-related risk factors associated with occurrence of PEP. Independent risk factors for PEP were assessed by multiple logistic regression and it showed age younger than 65 years old (*p* = 0.03, OR = 0.11) and EPBD (*p* = 0.011, OR = 20.35) were independent risk factors for PEP.

**Table 5:** Risk factors associated with PEP

	Univariate analysis		Multivariate analysis	
Factors	Odds ratio	P	Odds ratio	P
Age < 65	0.95 (0.9–1.0)	***0.05***	0.11 (0.02–0.80)	*0.03*
Gender	0.51 (0.1–2.3)	0.37		
NKF or TPS	3.5 (0.8–1.6)	0.18	11.57 (0.06–3.49)	0.45
No. p cannulation	1.3 (1.0–1.6)	0.03	1.4 (0.93–2,3)	0.09
PEP prophylaxis	1.6 (0.4–6.9)	0.53		
Diclofenac	10.7 (2.1–53)	0.004		
Gabexate mesilate	2.3 (0.5–10.2)	0.12		
P-stenting	0.9 (0.1–8.8)	0.97		
NSAIDs + FOY	16.5 (3.0–91.6)	0.001		
EPBD	7.75 (1.6–37.2)	0.01	11.57 (2.19–189.61)	0.008

*NKF* needle knife fistulotomy, *TPS* transpancreatic sphincterotomy, *EPBD* endoscopic papillary balloon dilatation, *PEP* Post-ERCP pancreatitis

## Discussion

Deep biliary cannulation is the critical step of endoscopic management for pancreatobiliary disease. Precut or pancreatic guidewire-assisted techniques are used when endoscopists encountered difficult biliary access. There were different criterias for difficult biliary access in several literatures and some recent consensuses [11–13]. In our study, these patients still met the criteria of the consensus for difficult biliary access. Furthermore, the criterias for difficult biliary access wouldn’t influence the success rate and complication rate of TPS and NKF. Literatures reported precut techniques achieve a high biliary access rate, and the initial success rates were 73.4 to 100% [1, 3, 14–16]. In our study, the biliary access rates were 74.2% for TPS and 83.% for NKF, which were similar to the published studies. NKF had higher biliary access rate, but there was not statistically significant. After salvage methods with precut techniques, the biliary cannulation rate increased from 93.6% (1408/1504) to 98.0%.

Our study found no difference between the two methods with regard to total complication rate, acute cholangitis and perforation. Post-ERCP hemorrhage is mainly described during or after sphincterotomy and the degree of hemorrhage may range from oozing to severe bleeding in up to 10 to 30% of cases [17–19]. Bleeding during pre-cut sphincterotomy was more frequently found in the NKF group than in the TPS group. However, bleeding usually stopped and there was no significant difference in bleeding between two groups according to the definition of consensus criteria. The published studies also support our findings [6, 15].

Difficult cannulation is considered to be an independent risk factor for PEP [20]. Using advanced cannulation techniques can increase the success rate for CBD cannulation, they also have the potential to significantly increase the adverse event rate. Unlike NKP and TPS, NKF does not involve the pancreatic orifice. An RCT demonstrated that NKF had a lower risk of PEP than NKP (0% vs 7.6%, *P* < .05) [3]. In a retrospective study NKF had a lower PEP rate than the NKP or TPS [14]. In our study, TPS group had higher Post-ERCP amylase level in the TPS group than in NKF group. PEP occurred more frequently in the TPS group thanthe NKF group, while the difference was not statistically significant. After adjusting possible confounders, we find age younger than 65-year-old and EPBD, but not biliary cannulation method, are independently associated with PEP.

Increased cannulation time, number of cannulation attempts, and number of pancreatic duct injections/cannulations have been associated with increased risk of PEP [21–23]. In TPS group, patients have more numbers of pancreatic cannulation and most of the patient (16/31) received three to six times of pancreatic duct cannulation during the procedure. This may result in higher post-ERCP amylasemia and higher PEP rate (although not statistically significant) in TPS group. In TPS group, patients with PEP had more pancreatic cannulation (PEP vs. non-PEP, median: 5 vs. 4, *p* = 0.21). Early pre-cut procedure maybe considered to prevent PEP when we plan to perform TPS.

There are several limitations in our study. This is a retrospective, single center study and the patient number in our study is small. Small sample size is a major limitation, and further large patient numbers of study in the future may be needed. Second, there was no uniform PEP prophylaxis in the presented study and it may affected the incidence of PEP. However, there were very few randomized studies comparing the efficacy and adverse event rates between TPS and NKF. Our experience provide additional real-world data on this issue.

## Conclusion

Both TPS and NKF have good biliary access rate in patient with difficult cannulation. TPS is a salvage technique with acceptable successful rate and complication rate for difficult biliary access. Younger age, and EPBD, but not biliary cannulation methods is associated with PEP in patient encountered difficult cannulation.

## Data Availability

The datasets used and/or analysed during the current study are available from the corresponding author on reasonable request.

## Abbreviations

TPS: Transpancreatic sphincterotomy
NKF: Needle knife fistulotomy
ERCP: Endoscopic retrograde cholangiopancreaticography
PEP: Post-ERCP pancreatitis
NKP: Needle knife papillotomy
RCT: Randomized control trials
CBD: Common bile duct
EPBD: Endoscopic papillary balloon dilatation
